# Comparison of frictional forces during the closure of extraction spaces in passive self-ligating brackets and conventionally ligated brackets using the finite element method

**DOI:** 10.4317/jced.55739

**Published:** 2019-05-01

**Authors:** Sandra-Liliana Gómez-Gómez, Natalia Sánchez-Obando, María-Antonia Álvarez-Castrillón, Yesid Montoya-Goez, Carlos M. Ardila

**Affiliations:** 1 Orthodontics; Master in Epidemiology; Assistant Professor, School of Dentistry, Universidad de Antioquia; 2 Orthodontics, Universidad de Antioquia; 3 Orthodontics. Universidad CES; 4 Civil Engineer. Master of Materials Science; Assistant Professor, Escuela de Ingeniería de Antioquia; 5 Periodontist. Ph.D in Epidemiology; Biomedical Stomatology Group, Universidad de Antioquia, Medellín, Colombia. Department of Periodontology, School of Dentistry, Universidad de Antioquia

## Abstract

**Background:**

This study compared the frictional force resulting from the bracket/archwire interface and the stress at the root/periodontal ligament/bone interface, between passive self-ligating brackets and conventionally ligated brackets, during the space closure stage.

**Material and Methods:**

A cone beam tomography was taken to a female patient that required extraction of upper first premolars and passive self-ligating system; three months after its activation, a cone beam tomography was taken again. The designs of the maxillary bone and the entire system were possible through tomography images and stereomicroscopic photographs. Validation of the Finite Element Method (FEM) was achieved comparing the amount of movement seen through tomography images and the FEM. Space closure was simulated for each system through the FEM and a comparison was made between the frictional force at the bracket/archwire interface, and the root/periodontal ligament/bone interface.

**Results:**

The most significant representation of frictional force at bracket/archwire interface and bone stress was found at the conventionally ligated system, while the passive self-ligating system accounted for the highest distribution of stress over the root.

**Conclusions:**

The FEM is an accurate tool used to quantify frictional force and stress concentration during the orthodontic closure. The passive self-ligating system was seen less frictional during the closure state compared to conventional brackets.

** Key words:**Friction, orthodontic bracket, finite element analysis.

## Introduction

Friction has been defined as the resistance to movement when an object is moved with respect to another one and it operates at the direction opposite to the movement on contact surfaces (1-4). In orthodontics, friction occurs after direct contact between bracket, arch wire, and ligature during sliding mechanics (4).

Friction gains orthodontic importance to create more appropriate stress systems for orthodontic dental movement with no damages to the periodontal tissues. A more efficient orthodontic dental movement and a better predictability of treatment can be achieved controlling the friction (1).

About 12% to 60% of the applied stress is used to exceed frictional force.2 This may require an excessive increase of the stress used in orthodontics, causing damage to supporting structures (2-4). Since the ligation method produces highly significant differences in friction, several modifications of brackets to control ligation stress and reduce friction, between archwire and slot have been created, integrating ligation systems such as the self-ligating brackets (5).

Despite prior studies have confirmed that self-ligating brackets generate less friction, during the alignment and leveling stage, compared to conventional brackets (1,6-8), sufficient evidence has not been found to assure that passive self-ligating brackets generate less friction that conventional brackets, during sliding mechanics at the space closure stage, when square and rectangular arches are used to increase contact area, then, it is assumed that it could increase such friction resistance(8).

The Finite Element Method (FEM) is a tool widely reported (9-14); it has a potential to predict, with significant accuracy (15-18). FEM consists of a mathematical model equivalent to a real object and it allows modeling complex geometric structures such as the teeth, periodontal ligament, and bone (13), facilitating the study of biological systems (9,14).

This study compared the frictional force resulting from the bracket/archwire interface and the stress at the root/periodontal ligament/bone interface, between passive self-ligating brackets and conventionally ligated brackets, during the space closure stage.

## Material and Methods

Considering that the finite element method is an exact mathematical model, it is enough to perform the analysis in a single patient (9,14). In a systemic and periodontally healthy patient with Class II malocclusion (15), protrusion of upper incisors, an orthodontic treatment was started with passive self-ligating brackets (standard Damon Q®-Torque). At the end of the alignment and leveling stage of maxillary arch, the following biomechanics was implemented for the space closure process.

The area from the second premolar to the second molar (posterior area), and between incisors and canines (anterior area) were consolidated with a continuous metal ligature under the arch. A stainless-steel archwire (0.019-inch x 0.025-inch) (Ormco, Orange, Calif) was inserted with pre-welded posts on the mesial side of the upper canines. The system delivered a stress of 100 g per side with NiTi 13-mm closed springs (Ormco, Orange, Calif), spreaded out from the first molar hook, and ligated to the pre-welded post with metal ligature.

Two cone-beam scans (CB) were taken of the upper jaw in T1 (before activating the closure system) and in T2 (three months after activation). The tomography study was conducted with the Orthopos XG5-3D equipment (Sirona, Siemens, Berlin, Germany). The distance between sections was 1 mm. The processing of images for future reconstruction was conducted with the Galileos Viewer software (Sirona, Vensheim, Germany).

-Construction of the Finite Element Model

Geometric Modeling 

Computer assisted design (CAD) of the closure systems were constructed using the SOLID-EDGE ST6 software, from pictures taken with stereomicroscope (Nikon Smz 1000) of passive self-ligating, conventional brackets (Minidiamond® slot 0.022-inch x 0.028-inch) and stainless-steel 0.019-inch x 0.025-inch arch wire (Ormco, Orange, Calif) with pre-welded posts on the mesial side of canines.

Based on the images obtained from the CB scan in T1, CAD reconstruction of the maxillary bone/teeth set, performing extrusion and casting operations was possible with the Solid-Edge St6 software; the periodontal ligament was modeled with a 0.2 mm thickness (10-12). Then, each bracket system (conventional and passive self-ligating) was assembled in an independent manner, according to the spatial distribution achieved with the CB scan, and then were generated in Parasolid files, then, they could be read.

Meshwork 

After the geometric modeling was completed in both systems, the next step was to generate the finite-element model, including the generation of a mesh from Parasolid files on the SolidWorks Simulation software. Regarding the passive self-ligating system (Damon Q®), each volume was meshed with a solid mesh based on the curvature, with high-degree quadratic elements and a total number of 2.553.474 nodes and a total number of 1.789.286 elements.

Concerning the conventionally system (Minidiamond®), each volume was meshed with a solid mesh based on the curvature, with high-degree quadratic elements and a total number of 936.516 nodes and a total number of 508.021 elements.

Simulation 

To adjust simulation to clinical reality, the following characteristics were included in the structures to be simulated:

• In the finite-element mesh of each system, loads and mechanical properties were applied to materials such as the Young’s modulus and the Poisson’s ratio (14,15) ( Table 1).

**Table 1:**
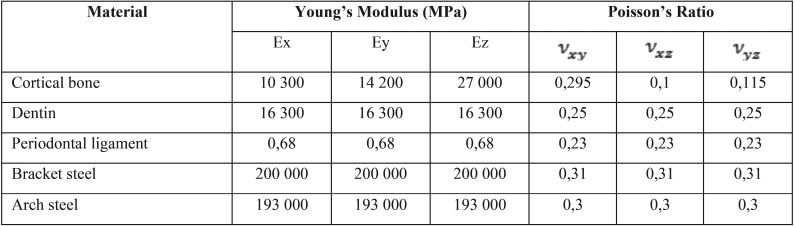
Mechanical Properties of Materials Used in Simulations.

• The alveolar bone and periodontal ligament were modeled as anisotropic rings (10,16), where mechanical properties may vary in the three planes of the space (ѵxy,ѵxz,ѵyz).

• Teeth, brackets, and dental arches were modeled as isotropic materials, where mechanical properties do not vary according to the space plane (17).

• Border conditions: restrictions were imposed to all degrees of freedom for the maxillary bone; additionally, consolidation was achieved through the assembly of anterior segment, canine to canine, and both posterior segments, from the second premolar to the second molar, simulating the action of a continuous metal ligature under the archwire.

• To obtain the real result of stress exercised by the NiTi spring on the patient, 100 g stress was administered with posterior direction from the pre-welded post, and 100 g stress was administered with anterior direction from the hook of the first molar.

• With the purpose of simulating the action of the elastic ligature on the conventionally system, a friction force of 0.061 newtons was added to each bracket (17).

• Because of the simulations, dental movement and compression forces were completed during the bone/tooth/periodontal ligament interface, expressed according to the Von Misses` criterion; cutting forces were also achieved during the bracket/arch wire interface, as the frictional resistance measurement.

-Validation 

**Figure 1:**
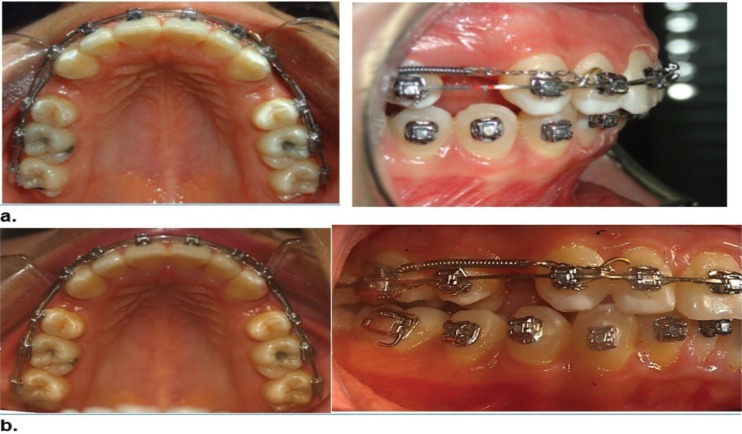
a. Clinical picture of upper jaw in T1; b. Clinical picture of upper jaw in T2.

The amount of dental movement was clinically measured with a digital gauge (Stainless Hardened); dental movement was also measured on the CB scan with the Galileo Viewer software, and it was defined by the space found between distal surface of upper canine and mesial surface of second premolar, in T1 and T2 (Fig. 1a,b).

In the tomography, longitudinal measures were taken to the space between canine and second premolar (in two planes of the space: tangential and axial), specifically in the section number 12 of T1 and T2, located 12 mm under a horizontal line, drawn from an anatomic reference point, defined in the distal surface of the root of the right upper second premolar. Also, the section number 12, in a tangential view of T1 and T2, angular measurements of mesio-distal inclination were taken, between longitudinal axis of tooth 15 and a vertical outlined perpendicular to the real horizontal defined in anterior sections.

After clinical and tomography measurements of space closure were obtained, they were compared to the results obtained with the FEM, to validate the method.

The study protocol was approved by the Institutional Review Board (IRB 2014-07). Also, an informed consent was obtained from the patient.

## Results

-Dental Movement 

**Figure 2:**
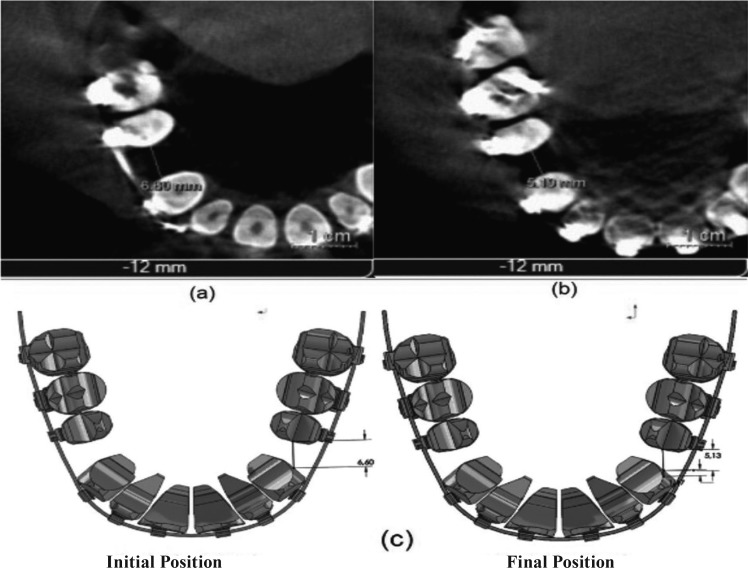
Dental movement in the occlusal plane with the passive self-ligating system. a. Distance measured in T1 through CAT scan. b. Distance measured in T2 through CAT scan. c. Amount of space closure measured through the FEM.

In the occlusal plane, the amount of space clinically measured from distal surface of tooth 13 and mesial surface of tooth 15 was 1.5 mm and 1.41 mm, respectively with the CAT scan (Figs. 1,2). Closure of space resulting from the FEM simulation was 1.47 mm for the passive self-ligating system and 1.3 mm for the conventionally ligated system; then, dental movement in the passive self-ligating system was 13% bigger that the one seen in the conventionally ligated system (Fig. 2). Simulation with the FEM showed evidence of dental rotation in teeth 15 and 25, as well as in teeth 12 and 22 in the passive self-ligating system, and no dental rotation was seen in the conventionally ligated system.

-Validation of the Finite Element Method 

According to the clinical measures obtained, the amount of space resulting from distal surface of tooth 13 to mesial surface of tooth 15, with the passive self-ligating system was 1.5 mm (Fig. 1); based on the tomography results, closure of spaces was 1.41 mm (Fig. 2), while dental movement shown with finite-element simulation was 1.47 mm (*p*=1) (Fig. 2).

Relating to the angular measurements taken in the tangential plane, the tomography showed a 12-degree angle of the right upper second premolar in T2; FEM simulation showed that the same angular measurement was 11.59-degree (*p*=1). Considering that there are no significant differences between dental movement, seen with tomography and FEM, it has been concluded that the numerical method simulates the real physical phenomenon.

-Frictional Resistance in Bracket/Archwire Interface 

**Figure 3:**
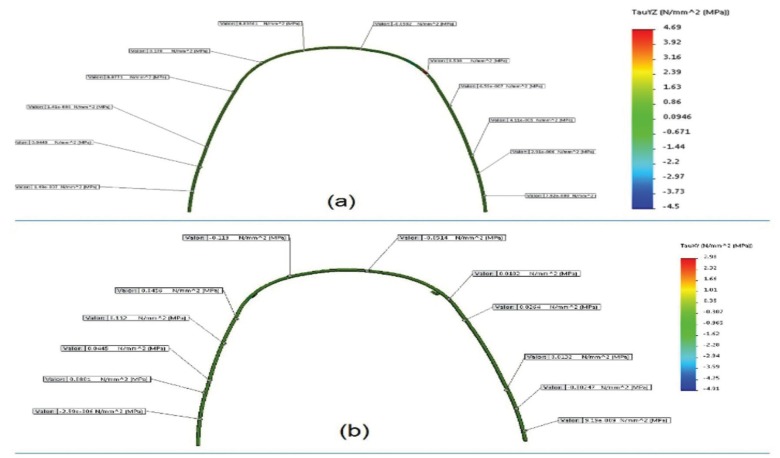
Graphic display of the cutting stress distribution (MPa) at the arch used in each System. a. Conventionally ligated system. b. Passive self-ligating system.

Comparing cutting forces, as a measurement of frictional resistance, a general tendency at specific points of the arch was not detected in any of the systems evaluated; however, the maximum cutting force was similarly found in the two types of dental system, on upper canines, with a stronger presence in the conventionally ligated system, with 4.69 MPa, compared to the passive ligation system, with 2.98 MPa; this accounts for 36.5% more of frictional resistance, in the conventional ligated system, compared to the self-ligating system (Fig. 3).

Stress in the Bone/Periodontal Ligament/Tooth Interface 

Maximum stresses on the bone according to the Von Mises’ criterion were of about 0.8319 mPa. The maximum stresses occurred on palatal, mesial, and distal surfaces of central incisors, and on cervical and palatal surface of canines. An asymmetry in the distribution of stresses, with a higher concentration on cervical, palatal, and distal surfaces of right upper second premolar, compared to the same surface on the left second premolar was observed. Maximum stresses on teeth were of about 1.3 MPa and occurred in all teeth, except for teeth 17 and 27, in the middle and apical thirds.

With respect to the conventionally ligated system, the maximum stresses on the bone ranged between 1.266 and 1.649 MPa. The maximum stresses occurred on the apical surface of teeth 21, 22, 23, and 13. Distribution of stresses showed an asymmetry, with a stronger stress on the cervical, palatal, and distal surfaces of tooth 15, compared to the same surface of tooth 25.

It should be noted that the periodontal ligament showed the same amount of stress as the bone; this is probably the result of its low density and the characteristic confined to the bone.

According to Von Mises’ criterion, the teeth showed a maximum stress between the middle and apical third, and half of their entire length, showing less stress on the area of molars (for both systems). Maximum stress was of 1.3 MPa in the passive self-ligating system, and 1.22 MPa in the conventional system, but a stress of 1.02 MPa predominated.

## Discussion

Several publications have shown the FEM as a tool to predict dental movement (13,17-19); however, these studies did not show a validation of the model; therefore, its real application seems to be inconclusive. When the FEM was applied, the variables with the supreme uncertainty were the mechanical properties of the bone, tooth, and periodontal ligament, not only for lack of studies in this field but related to its representativeness when is applied to a specific patient. In this study, however, a higher accuracy and adjustment to the biological reality analyzed can be shown when the FEM is validated, based on a real clinical model.

Based on the importance of validation for this type of methods, a comparison was completed between the amount of space closed with the FEM (1.47 mm) and the obtained with the tomography (1.41 mm) between T1 and T2, then, the movement could be equivalent to the clinical results, corroborating previous results (18); this may assure that the results obtained with the numerical simulations were adjusted to the real system.

Friction has been defined as the resistance to movement when an object is tangentially moved against another object, and it does it in a direction contrary to the displacement (2-4,6,8,20). As teeth move during an orthodontic treatment, frictional resistance occurs, impeding dental movement. It has been proven that the ligation method produces significant differences in the resulting friction (6). Several *in-vitro* studies have affirmed that friction in passive self-ligating brackets is lower that the conventionally ligated brackets (7,21-23); then, the most common thing to imply is that passive self-ligation improves clinical efficiency in movement mechanics. However, in this FEM-based study, a difference of 0.17 mm was found in the amount of space closure between passive self-ligating brackets and conventionally ligated brackets, which is not a clinically significant finding. This is consistent with clinical studies that compared the passive space closure, the passive space closure in a stage, and distalization of canines, between the types of brackets (24). Furthermore, there were no statistically significant differences in the movement efficiency. However, a systematic review (8) indicated that self-ligating brackets may generate less frictional force with rectangular arches, improving the movement efficiency, during a space closure.

Burrow (25) instead found that when individual retraction of maxillary canines is compared to conventionally ligated brackets and passive self-ligating brackets, in a split mouth design, the retraction speed was seen faster in conventionally ligated brackets, since the width of the conventional bracket is smaller than the passive self-ligating bracket; additionally, this study concluded that a narrower bracket generates higher momentums, and this increase sliding resistance.

It is important to accept that the critical phenomenon which determines orthodontic dental movement speed is the biological response of tissues to mechanical stresses exercised to move teeth (25); therefore, efficacy of treatment will depend on bone metabolism and periodontal ligament remodeling than on the interaction between bracket and ligature, in humans.

Friction generated in the bracket/arch wire interface tends to prevent expected movement. Ligation method is an important contributor to such friction force 4 and results in highly significant differences (5). During this study, it was found that friction in the bracket/archwire interface of conventionally ligated brackets was 36.5% stronger than in passive self-ligating brackets, which is consistent with previous results (3).

Several studies have found that if the archwire increases in size, also the frictional resistance increases (6,26). The main reason can be attributed to an increase of wire rigidity and contact surfaces (27). This study included space closure in both bracket systems, with a 0.019-inch x 0.025-inch steel arch; a stronger friction resistance was found in conventionally ligated brackets; this allows concluding that frictional resistance directly depends on the type of ligation and the type of bracket, much more than on the arch wire caliber. These results are contrary to previous results that reported no significant differences in frictional resistance between conventional brackets and passive self-ligating brackets, when combined with 0.019-inch x 0.025-inch rectangular wires (28). In a systematic review was concluded that passive self-ligating brackets combined with rectangular arches show less friction that conventionally ligated brackets (8). This study showed that rectangular arches, with a wider caliber, present the highest frictional resistance found in conventionally ligated brackets compared to passive self-ligating brackets. However, these results should be seen with precaution, because functional and environmental factors of the mouth have an influence on the movement mechanics (7). Food impacts during the mastication cause arch flexure and release of the bracket/archwire junction, which facilitates dental movement. It has also been observed that humidity conditions within the oral environment decrease frictional resistance (29). Therefore, *in vivo*, it is more likely to assume that the force required to release friction, it could be lower than the one measured in laboratory experiments. This research clarifies that frictional force in conventionally ligated brackets is directly related to elastic modules, which lose about 50% of initial force, within the first 24 hours, and then a decrease of 30% to 40% occurs, after four weeks (7). Hence, if the finite-element method has been widely applied to dental biomechanics (19), it should be considerate before making clinical decisions.

During this research, it was found that the strongest stresses in bone were seen in the conventionally ligated system with respect to the passive self-ligating system; this can be associated to the stronger frictional resistance seen in this system that will have a direct impact on the stresses supported by the osseous tissue.

In relation to the self-ligating system, this study corroborates a previous report; the strongest stresses were seen in the canine (disto-lingual direction) and in posterior teeth (mesio-lingual direction) (10). In the present study, the strongest stresses found in the self-ligating system include the palatal, mesial, and distal surfaces of central incisors and the cervical and palatal surface of canines, likely associated to the amount of force applied to the system, since during the study of Hortúa *et al.* (13) 150 g was applied per side, and only 100 g was applied in this study.

The strongest stresses on teeth were found at the middle and apical thirds in both systems, with stronger stresses seen in the passive self-ligating system; this is likely associated to the effectiveness found in dental movement, in the self-ligating system. The highest concentration of stress at the apical third in teeth and periodontal ligament and bone, of both systems, coincides with sites clinically associated to root reabsorption (30).

Despite the restrictions found in a mathematical model, it allows to conduct a representation of the frictional behavior between orthodontic systems, which facilitates the control of biological variables that may alter the clinical results.

## Conclusions

1. The FEM is a valid and reliable tool to predict dental movement expected during a treatment; however, mechanical properties of biological tissues should be carefully managed. Also, the FEM can be an effective tool to compare different orthodontic systems.

2. No clinically significant differences were found with respect to dental movement, during the space closure stage, between passive self-ligating brackets and conventionally ligated brackets.

3. During the bracket/archwire interface, the best representation of frictional resistance was conventionally ligated brackets, compared to passive self-ligating brackets.

4. The strongest stresses in bone tissue and periodontal ligament were found in the conventionally ligated system; the strongest stresses in root surface were found in the passive self-ligating system.
